# Perioperative Management of a Patient With Wolff-Parkinson-White Syndrome Undergoing Thyroidectomy With Bilateral Modified Neck Dissection and the Role of Dexmedetomidine: A Case Report

**DOI:** 10.7759/cureus.94165

**Published:** 2025-10-08

**Authors:** Sameen Ejaz, Hamood Ur Rehman, Asma Ashraf

**Affiliations:** 1 Anesthesia, Shaukat Khanum Memorial Cancer Hospital and Research Centre, Lahore, PAK

**Keywords:** dexmedetomidine, neck dissection, perioperative management, thyroidectomy, wpw syndrome

## Abstract

Wolff-Parkinson-White (WPW) syndrome is characterized by the presence of an accessory conduction pathway in the heart, predisposing patients to tachyarrhythmias and sudden cardiac death. The perioperative management of these patients poses a significant challenge to the anesthetist due to the increased risk of arrhythmias, particularly under anesthetic and surgical stress.

We report the successful perioperative management of a patient with WPW syndrome undergoing thyroidectomy with bilateral modified neck dissection. The nature of the surgery increased the risk of vagal stimulation, given the proximity of the carotid sinus to the surgical field. Careful anesthetic planning and intraoperative vigilance were essential to prevent complications. Strategies included careful selection of anesthetic agents, avoidance of sympathetic and vagal stimulation, and the use of dexmedetomidine to maintain hemodynamic and rhythm stability. This case contributes to the limited literature available on the anesthetic management of WPW syndrome in the context of thyroid surgeries with neck dissection.

## Introduction

Wolff-Parkinson-White (WPW) syndrome is a condition marked by the presence of at least one accessory conduction pathway in the heart. It predisposes the patient to conditions such as atrial and ventricular tachyarrhythmias, and even sudden cardiac death [1]. The accessory pathway, known as the “Bundle of Kent,” can conduct electrical impulses in an anterograde, retrograde, or bidirectional manner. When it conducts anterogradely, it is termed a “manifest” pathway and produces pre-excitation patterns on the electrocardiogram (ECG) [2].

The prevalence of WPW syndrome is estimated to be between 0.68 and 1.7 per 1,000, with males being more commonly affected [3]. Management of these patients perioperatively, during any surgical procedure, presents a significant challenge to the anesthetist, primarily due to the increased risk of arrhythmias triggered by surgical stress. Not only is improper treatment of the syndrome ineffective, but it can also precipitate rapid clinical deterioration and even cardiac arrest [4]. Therefore, meticulous care is essential when managing such patients.

In this case, an asymptomatic patient with WPW syndrome was successfully managed during total thyroidectomy with bilateral modified neck dissection using dexmedetomidine. Dexmedetomidine has demonstrated favorable effects in maintaining hemodynamic stability and minimizing rhythm disturbances. These effects may render it a valuable option for the perioperative management of patients with pre-excitation syndromes. However, further research is required to evaluate its efficacy and safety profile in such cases.

The nature of the surgery is also very important in such patients, as a modified neck dissection can put the patient at risk of vagal stimulation due to the proximity of the carotid sinus to the operating site. The carotid sinus is located at the bifurcation of the common carotid artery and is crucial in mediating the baroreceptor reflex. Unintentional carotid sinus massage can cause vagal stimulation, leading to adverse outcomes such as coronary artery syndrome, bradyarrhythmias, and even cardiac arrest [5].

A very limited literature is available on the perioperative management of patients with WPW syndrome. To our knowledge, this case report presents one of the first documented cases of anesthetic management in a patient with WPW syndrome undergoing total thyroidectomy with bilateral modified neck dissection.

## Case presentation

A 36-year-old female, American Society of Anesthesiologists (ASA) Class II, and a known case of medullary thyroid carcinoma, was scheduled for total thyroidectomy and bilateral modified neck dissection under general anesthesia. She was diagnosed with WPW syndrome type B incidentally on an ECG performed as part of a preoperative anesthesia assessment. Clinically, she was asymptomatic. There was no history of syncope, ischemic heart disease, hypertension, diabetes mellitus, or any other comorbid condition.

On examination, she was a young female of average build, with a body mass index of 19.5 kg/m². Her vital signs were normal, with a blood pressure of 121/76 mmHg and a pulse rate of 99 beats per minute. Airway examination revealed a Mallampati score of II, with adequate mouth opening and neck extension, and a thyromental distance of 6 cm. The systemic examination was unremarkable. She was clinically and biochemically euthyroid.

**Figure 1:**
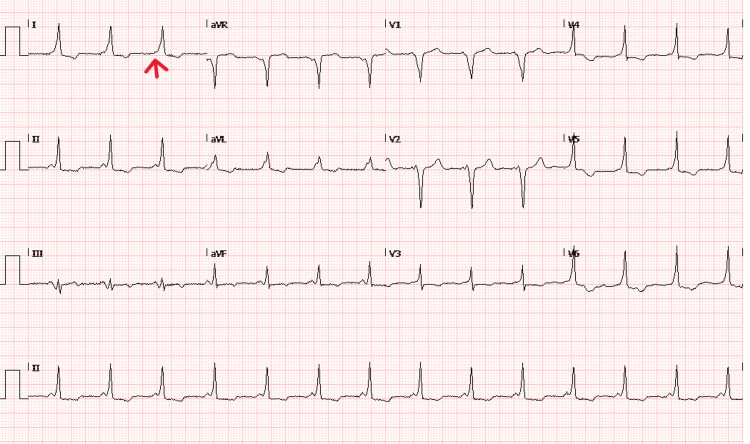
Preoperative Electrocardiogram of the Patient Demonstration of delta wave (red arrow) and short PR interval.

**Figure 2:**
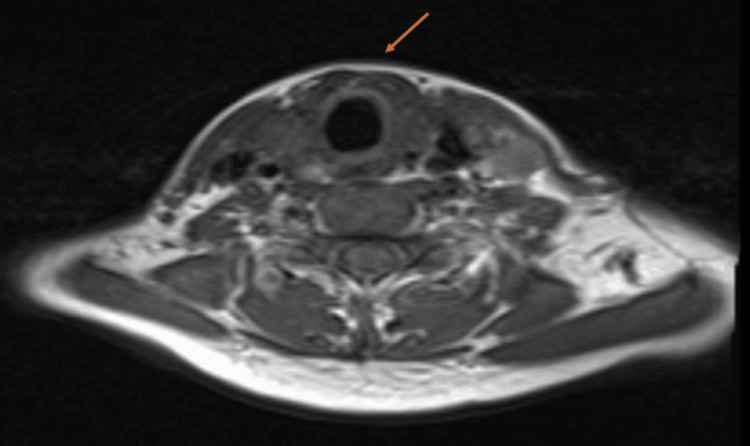
Axial Magnetic Resonance Imaging (MRI) of the Neck Without Contrast Demonstration of multiple T2 hyperintense nodules, predominantly in the left lobe and isthmus of the thyroid gland, as illustrated by the arrow. Mild tracheal displacement towards the right due to the mass effect of thyroid nodules on the trachea, but no tracheal invasion.

On investigation, the ECG showed a short PR interval, delta waves (slurred upstroke of the QRS complex), a wide QRS complex, and associated ST-T changes that led to the incidental diagnosis of WPW syndrome type B. There was pre-excitation on the resting ECG (Figure 1). A 2D echocardiogram revealed a normal study (no structural or functional defect), with an ejection fraction of 63%. Magnetic resonance imaging of the neck, as illustrated in Figure 2, showed an asymmetrically enlarged thyroid gland with multiple nodules primarily in the left lobe and isthmus, with internal areas of calcification and hyperintensities. The largest nodule measured 2.6 × 2.2 cm. There was no tracheal invasion, but a mass effect was seen on the trachea, with mild rightward tracheal deviation. There was no vascular or muscular involvement. Other lab investigations, including complete blood count, renal function tests, serum electrolytes, and thyroid function tests, were normal, as tabulated in Table 1. A 24-hour Holter monitoring was not performed, as it was not recommended by the cardiologist and was not available at our center.

**Table 1:** Patient's Baseline Preoperative Blood Tests and Reference Ranges Blood tests include complete blood count, renal function tests, serum electrolytes, and thyroid function tests. The reference ranges are according to the age and gender of the patient.

Parameter	Result	Normal Range	Units
Complete Blood Count:			
Hemoglobin	12.3	11-14.4	gram per deciliter
Hematocrit	39	33.3-44.6	%
Total leucocyte count	4.6 x 10^3^	4.52-10.93 x 10^3^	per microliter
Platelets	327 x 10^3^	150-450 x 10^3^	per microliter
Renal Function Tests:			
Urea nitrogen	8.13	8.0-19	milligram per deciliter
Creatinine	0.6	0.60-1.10	milligram per deciliter
Estimated glomerular filtration rate	113.16	more than 60	milliliter per minute per 1.73 square meter
Electrolytes:			
Sodium	136	136-145	millimole per liter
Potassium	4.15	3.5-5.1	millimole per liter
Chloride	106	98-107	millimole per liter
Thyroid Function Tests:			
Free T4 (tetraiodothyronine)	1.01	0.8-2.7	milligram per deciliter
Thyroid-stimulating hormone	2.177	0.4-4.2	micro international unit per milliliter

A cardiology consult was sought. The cardiologist deemed no treatment necessary for the patient preoperatively, owing to the asymptomatic nature of her pathology. However, postoperative follow-up in the cardiology clinic was planned. The patient was scheduled for surgery, and no treatment was started beforehand. The patient, along with her family, was counseled. A high-risk written informed consent was obtained from the patient after explaining all potential intraoperative complications. She was premedicated with oral alprazolam 0.5 mg on the night before surgery. A nil per os (NPO) period of six hours for solid food and two hours for clear liquids was ensured.

To ensure prompt management of arrhythmias, drugs such as amiodarone, lignocaine, adenosine, and inotropes were pre-arranged in the operating room (OR). A defibrillator was also kept ready in the OR for synchronized cardioversion, if needed. The patient was transferred to the OR, and standard monitoring was attached. The patient was injected with intravenous (IV) midazolam 2 mg and morphine 2 mg, and an awake arterial cannulation in the right radial artery was performed to enable invasive hemodynamic monitoring. An awake arterial cannulation was performed before induction so that beat-to-beat hemodynamic monitoring could be available during induction, and any sudden fluctuation in the blood pressure could be picked up and managed immediately. Two 18-gauge IV cannulas were also secured.

The patient was induced with IV propofol 150 mg, and muscle paralysis was achieved with atracurium 40 mg. The patient was ventilated for three minutes and then intubated with the help of a video laryngoscope, with a Fremantle score of 1. The definitive airway was secured with a reinforced endotracheal tube of size 7.0 mm internal diameter. At induction, there was a mild, transient increase in the heart rate and blood pressure of the patient, with no changes in ECG. This settled without any intervention.

**Figure 3:**
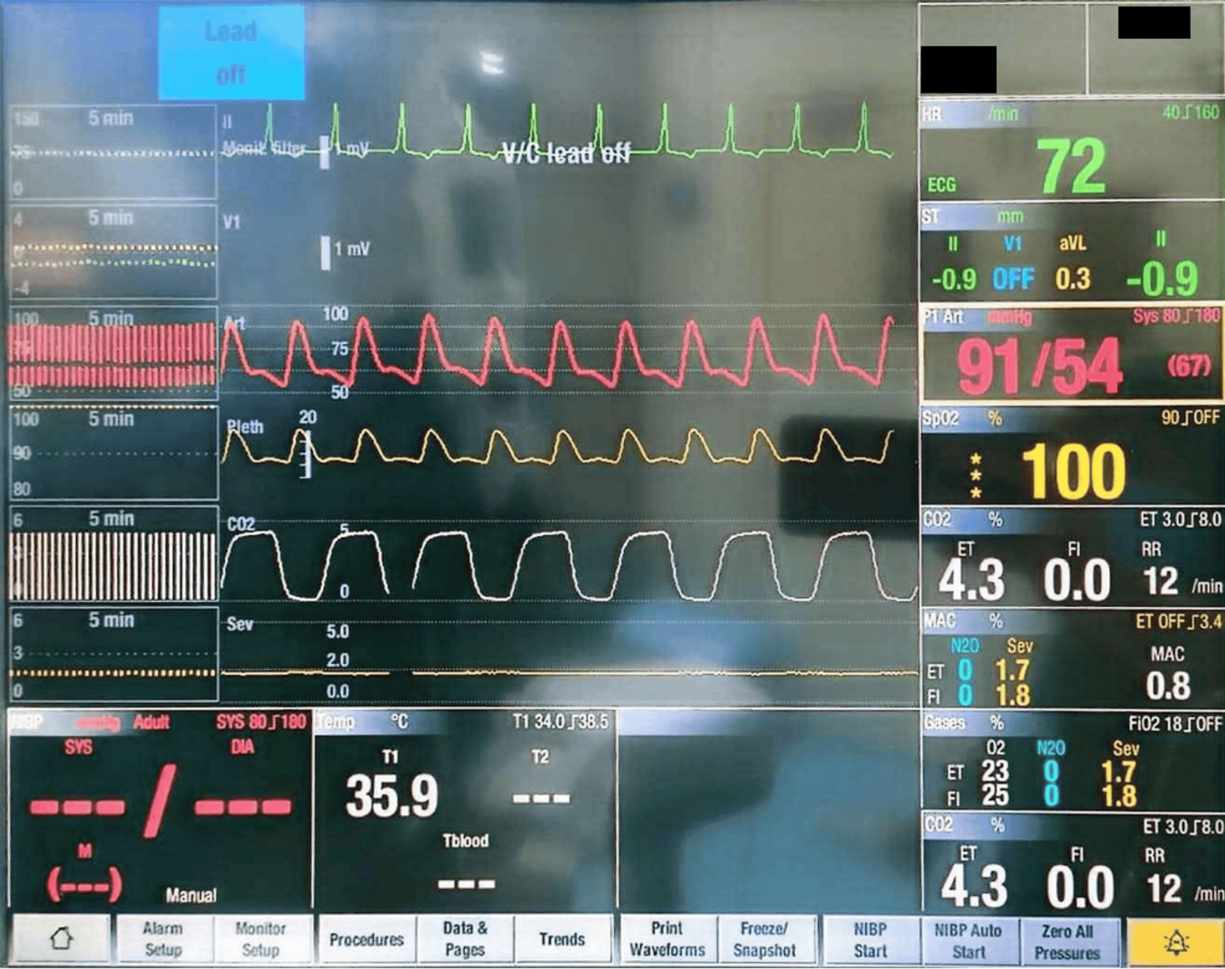
Intraoperative Monitoring of the Patient Showing Stable Hemodynamics Monitoring includes echocardiogram (ECG), heart rate, invasive blood pressure, oxygen saturation, end-tidal carbon dioxide, minimum alveolar concentration (MAC), and temperature. Note the delta wave in the ECG.

Anesthesia was maintained with oxygen, sevoflurane, and atracurium 6 mg as required. Adequate analgesia was ensured with IV paracetamol 1 g, IV diclofenac 75 mg, and IV morphine 4 mg. Before surgical incision, a dexmedetomidine infusion was started to maintain hemodynamic stability at a rate of 0.6 mcg/kg/hour and titrated according to the hemodynamic parameters - that is, a heart rate within 60-80 bpm and a mean arterial pressure (MAP) >65 mmHg - throughout the procedure. The intraoperative monitoring parameters are shown in Figure 3. The patient required intermittent boluses of phenylephrine (50 mcg each bolus) to keep the MAP above 65 mmHg. A good depth of anesthesia was maintained throughout the procedure - that is, a minimum alveolar concentration (MAC) of sevoflurane above 0.8%.

A closed-loop communication was maintained with the surgeons, especially during neck dissection and while performing the Valsalva maneuver. The Valsalva maneuver was performed twice to assist the surgeons without compromising hemodynamic stability. Sinus rhythm was maintained throughout the surgery. Arterial blood gas monitoring was also performed. Following completion of the surgery, deep extubation was carried out after confirming complete reversal of neuromuscular blockade. Complete reversal was ascertained by the appearance of four twitches in response to train-of-four stimulation of the ulnar nerve using a peripheral nerve stimulator. The airway was secured with a laryngeal mask airway (LMA) until the patient was fully awake, after which it was removed. Drugs such as neostigmine and glycopyrrolate were avoided to prevent any inadvertent bradyarrhythmia. Intraoperative prophylaxis for postoperative nausea and vomiting (PONV) was administered using IV metoclopramide 10 mg. The patient was then shifted to the post-anesthesia care unit (PACU) with strict monitoring of hemodynamic parameters, especially heart rate and rhythm. Postoperative analgesia was ensured with IV ketorolac 30 mg twice a day and IV paracetamol 1 g twice a day for three days, after which the patient was shifted to oral medications. Oral analgesics included paracetamol 500 mg three times a day and ibuprofen 400 mg twice a day. The subsequent course of recovery was uneventful, and the patient was discharged four days later. A cardiology follow-up, along with the surgical follow-up, was recommended for definitive treatment of WPW syndrome.

## Discussion

WPW syndrome is a form of ventricular pre-excitation characterized by the presence of an accessory pathway between the atria and ventricles, also known as the “Bundle of Kent” [6]. Normally, electrical impulses originate from the sinoatrial (SA) node and are then conducted through the atrioventricular (AV) node to depolarize the ventricles. If there is an additional pathway, as in WPW syndrome, it allows the impulse to bypass the AV node and directly reach the ventricles through the accessory pathway [7].

The initial diagnosis of WPW syndrome is based on the ECG pattern combined with symptomatology. The ECG findings consist of: (1) a short PR interval (<120 ms), (2) initial slurring of the QRS complex, known as the delta wave, (3) a wide QRS complex (>120 ms), and (4) secondary repolarization changes, visible as ST-T segment changes [8]. WPW syndrome may be symptomatic or asymptomatic. The American Heart Association guidelines recommend that asymptomatic patients with pre-excitation can be managed conservatively [9].

Anesthesia and surgery may unmask an undiagnosed syndrome; therefore, it is crucial not to miss the diagnosis. Diagnosed patients should undergo a detailed preoperative evaluation, focusing on the presence of signs and symptoms. They should be clinically optimized, if required. Cardiology consultation and close communication with the surgical team are cornerstones of a successful perioperative course in such patients. 

The anesthetic approach, encompassing the type of anesthesia, the choice of agents, and the technique employed, plays a pivotal role in the management of high-risk cases such as this, as these factors can significantly influence the physiological properties of cardiac conduction pathways. Preventive measures must be taken to attenuate the hemodynamic impact of anesthetic and surgical stimuli on the patient, as any disturbance in these parameters may lead to serious consequences, such as uncontrolled atrial fibrillation, supraventricular tachycardia (SVT), ventricular tachycardia, ventricular fibrillation, and sudden cardiac death.

The primary objective of anesthetic management in this patient was to minimize sympathetic stimulation throughout the perioperative period by addressing factors such as anxiety, pain, hypovolemia, a light plane of anesthesia, and the stress response associated with intubation and extubation. Additionally, all drugs with potential effects on cardiac conduction pathways were carefully avoided.

In this case, propofol was the preferred induction agent, as it does not affect the refractory period of the accessory pathway [10]. Vagolytics and other drugs that cause tachycardia, such as atropine, ketamine, and glycopyrrolate, were avoided. Sevoflurane was selected as the inhalational agent of choice because it has no effect on AV nodal conduction [11].

The choice of muscle relaxants is also critical in these patients. Succinylcholine should be avoided due to its arrhythmogenic properties [12]. Non-depolarizing muscle relaxants with better cardiac stability, such as rocuronium, vecuronium, and cisatracurium, are preferred over atracurium and pancuronium, as atracurium can cause histamine release leading to autonomic instability, and pancuronium may precipitate SVT [12].

Intraoperative episodes of hypotension were successfully managed with boluses of phenylephrine. Neostigmine, commonly used for the reversal of neuromuscular blockade, was avoided in this patient because it is not recommended in WPW syndrome, as it can enhance conduction through the accessory pathway [13]. The patient was ventilated until complete reversal of neuromuscular blockade was confirmed with the help of a peripheral nerve stimulator and then extubated.

Dexmedetomidine is an alpha-2 receptor agonist and a well-known analgesic, sedative, and anti-inflammatory drug, and recent studies have identified its anti-arrhythmic properties as well [14]. It exerts these effects by decreasing catecholamine release, prolonging the refractory period, and increasing vagal tone [14]. It has been successfully used to abort junctional and atrial tachyarrhythmias in recent case studies [15], and has also been shown to have cardioprotective effects when used perioperatively [16].

In this case, intraoperative administration of a dexmedetomidine infusion not only blunted the sympathetic surge associated with surgical stress and extubation but may have also played a role in maintaining rhythmic stability. Thus, it may be a good option in such patients, as it offers several advantages, including maintenance of anesthesia depth, hemodynamic stability, and perioperative analgesia.

Surgeries that involve the neck region increase the risk of arrhythmias. Any maneuver that increases vagal tone - such as unintended stimulation of the carotid sinus during neck dissection by the surgeons or the use of the Valsalva maneuver to assist surgeons - may slow conduction through the AV node and preferentially facilitate conduction via the accessory pathway, thereby amplifying the risk of arrhythmias [17]. Therefore, the surgical team was instructed to operate with great care in the neck region to avoid any inadvertent activation of the vagal pathway.

The Valsalva maneuver is commonly employed in thyroid and other head and neck surgeries to aid the surgeons in identifying bleeding sites. However, it can cause hemodynamic compromise by inducing bradycardia and hypotension due to activation of the baroreceptor reflex [18]. Though the Valsalva maneuver is often used to terminate arrhythmias in many cases of SVT, Nagappan et al. reported a case in which the Valsalva maneuver, used to terminate SVT in a patient with undiagnosed WPW syndrome, led to the development of atrial fibrillation and unstable ventricular fibrillation [19]. In this case, the Valsalva maneuver was performed twice intraoperatively to assist the surgeons in identifying bleeding sites, and no adverse events were observed.

## Conclusions

Patients with conduction abnormalities can be managed effectively when there is a thorough understanding of the underlying condition, the type of surgery, and the anesthetic plan. Special attention must be given to the choice of anesthetic agents, the avoidance of sympathetic and parasympathetic stimulation, and the maintenance of hemodynamic stability throughout the surgical procedure. The use of dexmedetomidine, an alpha-2 agonist, was a novel approach associated with favorable outcomes in this case. However, further research is required to validate its effectiveness. This case adds to the scarce literature available on the perioperative management of WPW syndrome in complex surgical settings.
